# Neuronal mechanisms of shift workers’ sleepiness

**DOI:** 10.1186/1471-2202-12-S1-F2

**Published:** 2011-07-18

**Authors:** Svetlana Postnova, Peter A Robinson

**Affiliations:** 1 School of Physics, University of Sydney, Sydney, New South Wales 2006, Australia; 2 Centre for Interdisciplinary Research and Understanding of Sleep (CIRUS), Woolcock Institute of Medical Research, University of Sydney, New South Wales 2037, Australia; 3 Brain Dynamics Centre, Sydney Medical School, University of Sydney, New South Wales 2145, Australia

## 

In an attempt to use all 24 hours of the day, shift work is becoming increasingly popular. Long term shift work leads to multiple health problems, including higher risk of cardiovascular diseases, mood disorders, and diabetes. Other consequences include loss of concentration and increase of sleepiness resulting in accidents [1]. Given the prevalence of shift work and the severity of the associated hazards, understanding of the mechanisms underlying sleepiness and predictions of shift workers’ fatigue would prove highly valuable. A number of mathematical models addressing these questions exist in a current literature. However, most of them are phenomenological and are limited to studying short-term effects of shift work; i.e., sleepiness only during the first couple of days on the shift [2].

**Figure 1:**
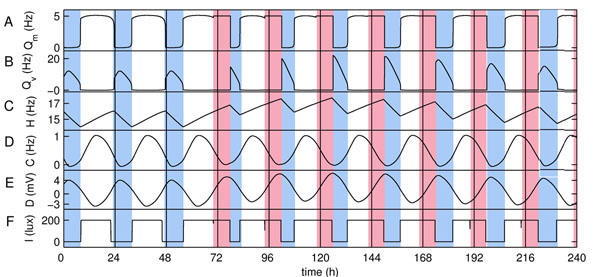
Sleep-wake activity with and without shift work. Permanent shifts are introduced on the 3rd day of the simulation between 22.00 and 06.00. Variables shown on the plot are: mean firing rate across the neurons in (A) monoaminergic and (B) ventrolateral preoptic nuclei, (C) homeostatic sleep drive, (D) circadian process, (E) total sleep drive, and (F) intensity of light to which the worker is exposed.

The model that is used here follows a different, physiologically-based approach, combining a quantitative model of sleep-wake switch [3] with a model of the human circadian pacemaker entrained by light and non-photic inputs [4]. This model accounts for the state-related transitions in the firing of wake-active monoaminergic (Fig.1A) and sleep-active ventrolateral preoptic nuclei in the brain (Fig.1B) under the influence of homeostatic and circadian drives shown in Fig.1C and Fig.1D . Homeostatic drive is responsible for accumulation of sleep pressure during wakefulness, while the circadian drive, which is controlled by the suprachiasmatic nucleus of the hypothalamus, provides a 24 hour periodicity of sleep-wake cycles, and is entrained by external light/dark cycle (Fig.1F) and non-photic stimuli. During shift work (pink areas in Fig.1) external cues, as well as sleeping times (blue areas in Fig.1) are changed, affecting the total sleep drive and sleepiness of shift workers (Fig.1E).

Using this model the physiological mechanisms responsible for shift-related changes in sleepiness are examined in the simplest case of permanent shift work. In good agreement with experimental data sleepiness was shown to increase during the first days on the evening, night and early morning shifts. This is explained by the inability to sleep enough during the active circadian phase and the thereby increased homeostatic pressure. After this initial increase, sleepiness decreases, and stabilizes due to circadian entrainment to the new external cues provided by the shifts. The entrainment time and the degree of sleepiness are higher for the shifts leading to a stronger change of the circadian phase comparing to the no-shift situation. The performance of shift workers was shown to be improved by increasing lighting intensity at work place and by decreasing light during breaks. Altogether, this model has shown to be a powerful tool for the research of mechanisms of sleepiness, and for design of optimal shift schedules.
